# Two *RECK* Splice Variants (Long and Short) Are Differentially Expressed in Patients with Stable and Unstable Coronary Artery Disease: A Pilot Study

**DOI:** 10.3390/genes12060939

**Published:** 2021-06-19

**Authors:** Chiara Vancheri, Elena Morini, Francesca Romana Prandi, Elie Alkhoury, Roberto Celotto, Francesco Romeo, Giuseppe Novelli, Francesca Amati

**Affiliations:** 1 Genetics Unit, Department of Biomedicine and Prevention, University of Rome “Tor Vergata”, 00133 Rome, Italy; chiara.vancheri88@gmail.com (C.V.); morinielena.em@gmail.com (E.M.); gnovelli@me.com (G.N.); 2 Unit of Cardiology, University Hospital “Tor Vergata”, 00133 Rome, Italy; francescaromanaprandi@gmail.com (F.R.P.); elie.alkhoury22@gmail.com (E.A.); celott.roberto@yahoo.it (R.C.); romeocerabino@gmail.com (F.R.); 3 Unicamillus International Medical University, 00131 Rome, Italy; 4 Medical Genetics Laboratories, Tor Vergata University Hospital, PTV, 00133 Rome, Italy; 5 Neuromed IRCCS Institute, 86077 Pozzilli, Italy; 6 School of Medicine, Reno University of Nevada, Reno, NV 1664, USA; 7 Department for the Promotion of Human Science and Quality of Life, University San Raffaele, 00166 Rome, Italy

**Keywords:** *RECK*, alternative splicing event, splicing variants, biomarkers, coronary artery disease

## Abstract

Primary prevention is crucial for coronary heart disease (CAD) and the identification of new reliable biomarkers might help risk stratification or predict adverse coronary events. Alternative splicing (AS) is a less investigated genetic factors implicated in CAD etiology. We performed an RNA-seq study on PBMCs from CAD patients and control subjects (CTR) and observed 113 differentially regulated AS events (24 up and 89 downregulated) in 86 genes. The *RECK* (Reversion-inducing-cysteine-rich protein with Kazal motifs) gene was further analyzed in a larger case study (24 CTR subjects, 72 CAD and 32 AMI patients) for its Splicing-Index FC (FC = −2.64; *p* = 0.0217), the AS event involving an exon (exon 18), and its role in vascular inflammation and remodeling. We observed a significant downregulation of Long *RECK* splice variant (containing exon 18) in PBMCs of AMI compared to CTR subjects (FC = −3.3; *p* < 0.005). Interestingly, the Short *RECK* splice variant (lacking exon 18) was under-expressed in AMI compared to both CTR (FC = −4.5; *p* < 0.0001) and CAD patients (FC = −4.2; *p* < 0.0001). A ROC curve, constructed combining Long and Short *RECK* expression data, shows an AUC = 0.81 (*p* < 0.001) to distinguish AMI from stable CAD patients. A significant negative correlation between Long *RECK* and triglycerides in CTR group and a positive correlation in the AMI group was found. The combined evaluation of Long and Short *RECK* expression levels is a potential genomic biomarker for the discrimination of AMI from CAD patients. Our results underline the relevance of deeper studies on the expression of these two splice variants to elucidate their functional role in CAD development and progression.

## 1. Introduction

Cardiovascular diseases (CVDs) are a group of pathologies of the heart and blood vessels that include coronary heart disease (CAD) and its main complication, acute myocardial infarction (AMI) [1]. CVDs are the leading cause of global mortality and disease burden in the world. CVDs cases prevalence was 523 million in 2019 and the number of CVDs deaths are 18.6 million each year [1]. CAD is related to the progressive development of atherosclerotic plaques in blood vessels that lead to the onset of atherosclerosis [2]. Atherosclerosis is a chronic inflammatory vascular process beginning with endothelial cells dysfunction. This process can lead to the obstruction of the vessels through accumulation of fat and cholesterol in the lumen of arteries causing AMI [2,3,4]. CAD is a multifactorial disease, with genetic/epigenetic and acquired factors implicated in its etiology. Among the genetic factors, alternative splicing (AS), a physiological mechanism that produces different arrays of messenger RNA (mRNA) isoforms from a single primary transcript, arises as a crucial event regulating complex biological processes [5,6,7,8]. Alternative splicing was first reported in 1977 for adenovirus mRNA [5,6,9]. AS is the process that produces proteins with different function, structure, localization and regulation; indeed, the exons can be in- or excluded from the transcript in different combinations to produce different functional RNA transcripts from a single gene [8]. It is estimated that 86% of all genes are alternatively spliced [10]. Seven different types of alternative splicing events are known: exon jump (also known as exon cassette), mutually exclusive exon, alternative donor (5′ splice) site, alternative acceptor (3′ splice) site, intron retention, alternative promoters and alternative polyadenylation site [11]. AS has an important role during cardiac development; alterations of AS carry on to progression of various heart-related diseases including cardiomyopathies, arrhythmias, and other cardiac pathologies [7,12,13]. Alterations in sarcomeric gene splicing were among the first to be related to acquired and inherited heart disease [12,14,15]. Indeed, in the heart, AS of exon 2b of *TPM1* gene (Tropomyosin α 1) generates a splice variant associated with human dilated cardiomyopathy (DCM) and heart failure [16]. It is demonstrated that mice overexpressing this splice variant in the heart manifest an impairment of cardiac function, a decrease of Ca^2+^ sensitivity in myofilament and DCM [16]. Calcium entry through the cell membrane is regulated by voltage-dependent L-type calcium channels composed of α1 (pore) and α2/δ and β subunits, plus, for some channels, a γ subunit in a ratio 1:1:1:1. In the heart, the α1 subunit (Ca_V_1.2) undergoes AS in various exons both during physiological development and in heart failure [17]. Cardiac and smooth muscle express distinct *Ca_V_1.2* splice variants and mutations of *Ca_V_1.2* have different functional impact depending on which exon is involved in AS events [18].

RECK, Reversion-inducing-cysteine-rich protein with Kazal motifs, is a glycoprotein of 971 amino acid residues (125 kDa) anchored to the membrane through a C-terminal glycosylphosphatidylinositol (GPI) modification [19]. RECK is a membrane-anchored matrix metalloproteinases (MMP) inhibitor and plays a role in inflammation [20]. The binding between RECK and MMPs, reduces MMPs activity, changes extracellular matrix proteins (ECM) levels and alters cell motility [21]. Through the bound with different MMPs, RECK glycoprotein has a key role in cellular migration processes and in the remodeling of extracellular matrix [22,23]. Moreover, RECK is considered a multifunctional protein involved in the creation of tissue microenvironment, not only by directly inhibiting MMPs activity, but also, by modulating different cellular pathways as Notch and Wnt pathways and thus playing a role in cell commitment to specific differentiation fates [24]. It has been shown that *RECK* gene is subjected to alternative splicing events. Five different alternative transcripts are in the NCBI database: the full-length transcript *RECK* (in this paper named Long *RECK*, transcript variant 1; NM_021111); the transcript variant 2 (NM_001316345); the transcript variant 3 (NM_001316346); the transcript variant 4 (NM_001316347) and the transcript variant 5 (NM_001316348, named in this paper Short *RECK*). Functional data related to transcript variants 3 and 5 demonstrated they hamper some of the anti-migratory and anti-growth effects of the Long RECK [20,21]. Short *RECK* shows a distinct sequence from exon 8 until C-terminus than the other splice variants; moreover, in place of the last 13 exon, it has an alternate 3′-most exon and thus results with a shorter and different C-terminus [25,26]. It has been demonstrated that Short RECK interacts with Long RECK through Kazal Motif, located from amino acids 635 to 773, and thus altering MMPs-Long RECK interaction and consequently the release of MMPs [21]. An increased expression and/or activation of MMPs and alteration of ECM components are associated with the pathogenesis of cardiovascular diseases [27]. The expression and regulation of Long and Short *RECK* splice variants in healthy and diseased heart and vasculature is currently not well known and warrants deeper studies.

In this study, we investigated the AS pattern in peripheral blood mononuclear cells (PBMCs) of subjects with absence of atheromatous plaques in coronary arteries (CTR group) and in patients with stable and unstable coronary artery disease (CAD and AMI groups, respectively) and identified a significant differential expression of Long and Short *RECK* splice variants. This expression study suggests that alternative splice variants may have an important role as a biomarker of CAD and that a deeper evaluation of their functional role in atherosclerosis may be important to unravel the molecular mechanism underlying plaque vulnerability.

## 2. Materials and Methods

### 2.1. Participants’ Enrollment and Samples Collection

We recruited 128 patients from January to December 2019; 24 control subjects (CTR group), 72 patients with stable coronary artery disease (CAD group) and 32 patients arrived at the attention of the Unit of Cardiology (“Tor Vergata” Polyclinic of Rome) during a myocardial infarction event (AMI group). A peripheral blood sample (9 mL) has been collected within 24 h from the coronary angiography for CAD and CTR group and within 24 h from the onset of myocardial event and after percutaneous coronary intervention (PCI) for AMI group (dx.doi.org/10.17504/protocols.io.zpvf5n6 accessed on 14 June 2021).

All 128 patients underwent coronary angiography to assess the presence and degree of coronary artery lesions. CTR group includes subjects without angiographically significant coronary artery stenosis. Significant coronary artery disease is defined by invasive coronary angiography as >50% stenosis of the left main stem, >70% stenosis in a major coronary vessel, or 30% to 70% stenosis with fractional flow reserve ≤0.8 [28]. Subjects in the control group were treated with medical therapy in presence of non-hemodynamically significant coronary artery stenosis. CAD group includes individuals with chronic stable angiographically documented coronary artery disease, admitted to the hospital after a positive provocative cardiac test for inducible myocardial ischemia. Patients in the CAD group underwent coronary angioplasty if hemodynamically significant coronary artery lesions were found. AMI group includes patients arrived at the emergency room (ER) with signs or symptoms of acute myocardial ischemia and diagnosed with ST segment elevation or non-ST segment elevation acute myocardial infarction, according to the universal definition of acute myocardial infarction [29]. In the AMI group, primary PCI was the procedure performed initially to treat the culprit lesion; any other hemodynamically significant lesions were treated during the same procedure or in a staged coronary intervention.

Inclusion criteria were: (a) gender (especially male); (b) age > 30 years; (c) dyslipidemia (total cholesterol ≥ 240 mg/dl, LDL-c ≥ 160 mg/dl, HDL-c < 40 mg/dl, Triglycerides ≥ 200 mg/dl) [30]; (d) hypertension, systolic blood pressure (BP) values ≥ 140 mmHg and/or diastolic BP values ≥ 90 mmHg [31]; (e) diabetes, fasting plasma glucose ≥ 126 mg/dl (7.0 mmol/L) or 2-h plasma glucose during 75 mg oral glucose tolerance test ≥ 200 mg/dl (11.1 mmol/L) or Hb1Ac ≥ 6.5% (48 mmol/mol) [32]; (f) smoking history.

Patients aged <30-year-old, or with heart failure, neoplastic disease, autoimmune disease, inflammatory chronic disease, chronic kidney disease (creatinine clearance < 15 mL/min) and a previous medical history of acute myocardial infarction have been excluded from this study.

The Ethics Committee for Clinical Research of the “Tor Vergata” Polyclinic of Rome approved this study (PROTOCOL OF STUDY REGISTER OF EXPERIMENTS 30/15).

### 2.2. PBMCs Isolation

PBMCs have been isolated by whole blood (9 mL) using Ficoll^®^ Paque Plus (GE Healthcare, Little Chalfont, UK) according to Rizzacasa et al. [2].

### 2.3. RNA Sequencing Study

RNA sequencing study has been performed on two patients of CTR and CAD group, matched for age and clinical features. RNA extraction and evaluation were performed as described [2]. RNA sequencing has been performed in collaboration with the “ICM-Plateforme de Génotypage-Séquençage” (Hôpital de la Pitié Salpêtrière, Paris, France) and GenoSplice (www.genosplice.com (accessed on 14 June 2021)). For sequencing, 500 ng of fresh RNA input extracted from PBMCs have been used. For library preparation, KAPA mRNA HyperPrep Kit for ILLUMINA (San Diego, CA 92122, USA) has been used. Sequencing has been performed using NextSeq 500 ILLUMINA platform. All samples passed quality control analysis.

### 2.4. RNA Sequencing Data Analysis

FastQC (Version 0.11.9), Picard-Tools (a set of command line tools for manipulating high-throughput sequencing (HTS) data and formats such as SAM/BAM/CRAM and VCF), Samtools (a suite of programs for interacting with high-throughput sequencing data) and RSeQC package (v3.0.1) were used for sequencing, data quality, reads repartition (e.g., for potential ribosomal contamination) and insert size estimation. Reads were mapped on the hg19 Human genome assembly using STARv2.4.0 [33]; Briefly, for gene expression regulation study, for each gene present in the Human FAST DB v2016_1 annotation, reads aligning on constitutive regions (that are not prone to alternative splicing) were counted. Based on these read counts, normalization and differential genes expression were performed using DESeq2 [34] on R (v.3.2.5). Only genes expressed in at least one of the two compared experimental conditions were further analyzed. Genes were considered as expressed if their rpkm (Read Per Kilobase per Million mapped reads) value was higher than 97.5% of the background rpkm value based on intergenic regions. Results were considered statistically significant for uncorrected *p* ≤ 0.05 and fold-changes ≥ 1.5.

### 2.5. Regulated Patterns-Exons Analysis

In order to analyze the different expression level of specific splice variants of known human genes in each sample of the two groups (CTR and CAD), the alternative splicing events and the gene structure were analyzed using EASANA^®^ GenoSplice (www.genosplice.com (accessed on 14 June 2021)) software which is based on FAST DB database (Friendly Alternative Splicing and Transcripts Database). EASANA^®^ is a bioinformatics solution dedicated to genome-wide gene expression studies using microarrays and Next Generation Sequencing (NGS), while FAST DB is a website resource for the study of the regulation of the expression of human gene products. FAST DB allows one to define the exons content of the different known transcripts produced by a human gene, based on a computerized analysis of human and mouse cDNAs and ESTs (human expressed sequence tags) libraries. In addition, it performs a multi-alignment of all the transcript sequences of a specific gene that is necessary for visualizing the common and specific sequences of these transcripts [35]. The FAST DB algorithm recovered all the exon sequences defined in Ensembl version 26 (homo_sapiens_core_26_35 database) and each of these exons was “blasted” against two cDNA databanks using standalone BLAST v2.2.10. A full-length transcript databank was downloaded from the UCSC website (genome.ucsc.edu (accessed on 14 June 2021)) and a partial mRNA databank was downloaded from the NCBI website (www.ncbi.nlm.nih.gov (accessed on 14 June 2021)). To define alternative events generating the different products of a single gene, the FAST DB algorithm compared each “transcript exon” with its corresponding “genomic exon”. FAST DB defined different types of events. An alternative first exon indicates that a “transcript exon” has to be the first exon of at least one transcript and, if there are other internal exons at this genomic position, it had to start at least 10 nt upstream of the first position of the corresponding “genomic exon”. To be defined as an alternative last exon, a “transcript exon” has to be the last exon of at least one transcript and, if there are other internal exons at this genomic position, it had to end at least 10 nt downstream of the last position of the corresponding “genomic exon”. An “alternative 3′-splice site” event is defined when the first position of a “transcript exon” is different from the first position of the corresponding “genomic exon”. An “alternative 5′-splice site” event is defined when the last position of a “transcript exon” is different from the last position of the corresponding “genomic exon”. An “intron retention” event delineates when a whole intronic sequence is included in at least one “transcript exon” sequence. “Exon skipping” event indicates when at least one transcript has not defined “genomic exon”.

For a given gene, the sequences of “transcript exons” corresponding to one “genomic exon” were aligned using Clustalw. The results for each “genomic exon” positions were then assembled to present the multi-alignment of the different cDNAs.

For each exon or intron involved in these events, we have verified that they passed the “yellow horizontal bar” on EASANA^®^ technology that shows the expression level of the exon/intron involved in the alternative splicing event and the regulation of these (upregulated and downregulated) between the two groups of the comparison. We also verified that the alternative splicing event indicated for each exon or intron involved a known transcript and not an artificial construct.

### 2.6. RECK Splice Variants Analysis by qRT-PCR

For mRNA fraction, 1.5 γ of total RNA was reverse transcribed into cDNA using the High-Capacity cDNA Reverse Transcription Kit (Applied Biosystems, Waltham, MA, USA) according to manufacturer’s instructions. Evaluation of Short and Long *RECK* splice variant was conducted by qRT-PCR with ABI7500 Fast Real-time PCR System using Power Sybr Green (Life technologies) and specific primer pairs (Table 1). To ensure the specificity of the qRT-PCR assays, primers design met several criteria:

1. Primer pairs must be designed to discriminate between the two splice variants (Long and Short) of the same gene. Indeed, we have aligned the sequences of the five transcripts using the Clustal Omega software (www.ebi.ac.uk (accessed on 14 June 2021)) and selected the diversity between them, therefore each of the two primers pairs used are specific to amplify the corresponding transcript. For the Long splice variant (transcript variant 1) the primer pairs were designed with the forward (Fw) primer between the exons 17–18 while the reverse (Rev) primer was designed between the exons 18–19 in order to amplify the exon 18 involved in the AS event. For the Short splice variant (transcript variant 5) the primer pairs were designed with the forward (Fw) primer in exon 8 (whose sequence is common between the five transcripts) while the reverse (Rev) primer was designed in exon 9 (whose sequence is specific for this variant 5, Supplementary Figure S1);
2. PCR product size must be about 100–230 bp;
3. An evaluation on Ensembl Genome Browser (https://www.ensembl.org/index.html (accessed on 14 June 2021)) excluded common SNPs in the primers. Finally, another general requirement considered was the similar annealing temperatures and a balanced G/C content.

We used *GAPDH* for data normalization and analysis of the results. Data analysis was performed using the comparative threshold cycle (Ct) method quantification as describe in Tiano et al. [36].

### 2.7. Statistical Analysis

Statistical analysis was performed using GraphPad Prism 6.0 (GraphPad Software, San Diego, CA, USA) and SPSS program, version 19 (IBM Corp, Amonk, NY, USA). The distribution of expression data was analyzed by Kolmogorov–Smirnov test. Mann–Whitney test, Wilcoxon test and Kruskal–Wallis test were used for data analysis when appropriate. For parametric and non-parametric distribution, expression data are represented as mean ± SD (standard deviation). Clinical differences have been analyzed using a Student’s *t*-test and data are represented as mean ± SD. For all analysis, significance was set at *p* ≤ 0.05.

## 3. Results

### 3.1. RNA Sequencing Study

In order to identify pathogenic AS events related to atherosclerosis and putative biomarkers able to discriminate between subjects with free coronary arteries (CTR subjects) and patients with coronary artery disease (CAD patients), we performed an RNA sequencing study on PBMCs of two patients of each group matched for age and clinical features (Supplementary Table S1).

All the selected subjects were male with an age >70 years old (CTR median age was 70.5 ± 11.1 years old, while CAD median age was 71.5 ± 4.9). Arterial hypertension and dyslipidemia affected both groups. Only CAD patients showed a smoking history (100% of CAD were ex-smokers). One CAD patient (50%) had type 2 diabetes mellitus.

**Figure 1:**
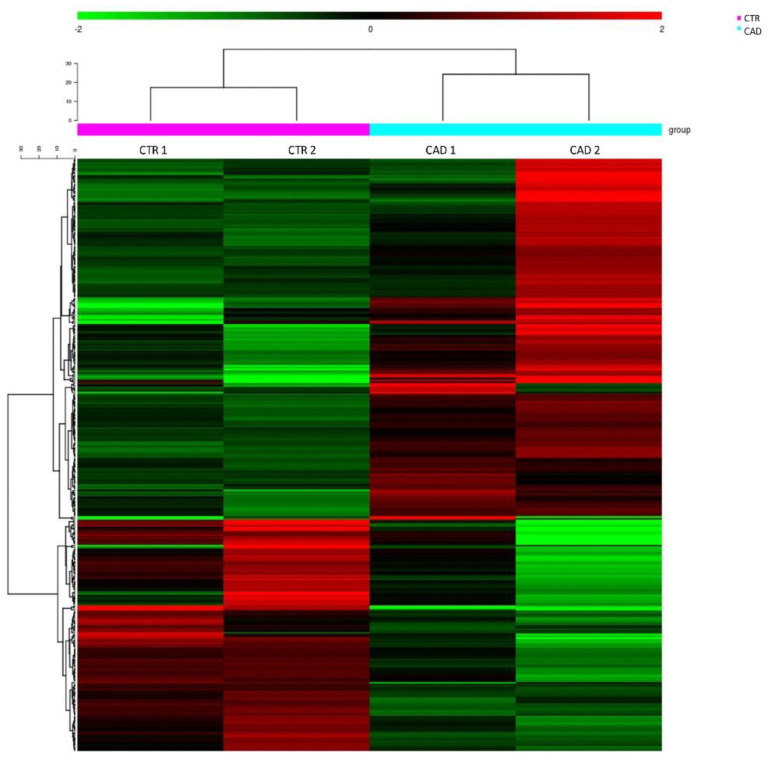
Heat Map resulting from RNA sequencing in the comparison coronary artery disease (CAD) vs. control subjects (CTR). The Heat Map showed a clear similar mRNA expression pattern between patients of the same group of study and a profile considerably different between each category of patients. In green are represented the downregulated mRNAs compared to the group set as control; in red are represented the upregulated mRNAs compared to the group set as control.

Sequencing has been performed on RNA extracted from PBMCs derived from these four samples. Cluster analysis of the RNA sequencing data indicated a peculiar RNA expression profile in each group of patients and revealed a clear separation of CTR and CAD patients (Figure 1).

**Figure 2:**
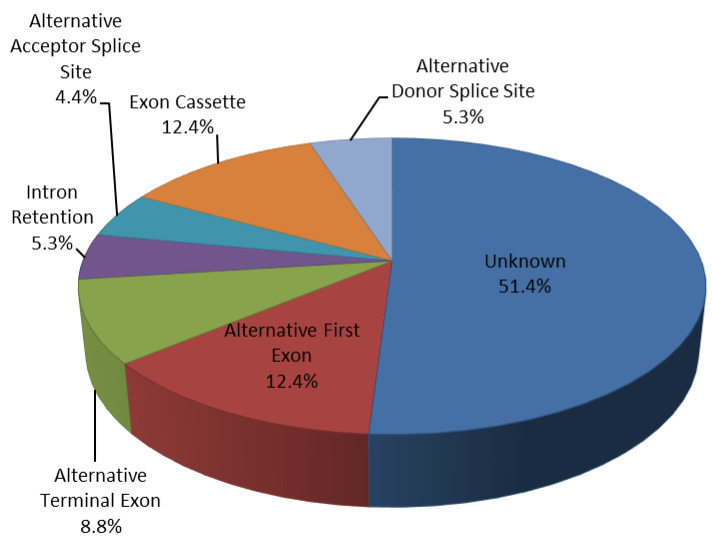
Alternative splicing events. Percentage of 113 differentially regulated alternative splicing events from 86 distinct genes: 4.4% Alternative Acceptor Splice Site; 12.4% Exon cassette; 5.3% Intron retention; 5.3% Alternative Donor Splice Site; 8.8% Alternative Terminal Exon; 12.4% Alternative First Exon; 51.4% unknown.

RNA-seq results showed differentially alternative splicing events (AS) in CAD PBMCs. Filtering for a statistically significant Splicing-Index Fold-Change (SI-FC ≥ ±1.5), we observed 113 differentially regulated alternative splicing events (24 upregulated and 89 downregulated) in 86 distinct genes (Figure 2 and Table 2).

These AS events were 14 alternative first exon events (12.4%), 10 alternative terminal exon events (8.8%), 14 exon cassette events (12.4%), 5 alternative acceptor splice sites events (4.4%), 6 alternative donor splice sites events (5.3%), 6 intron retention events (5.3%) and 58 unknown events (51.4%) (Figure 2 and Table 2). Selecting a SI-Fold-Change ≥ ±2 we identified 10 differentially regulated AS events (27% exon cassette, 27% alternative terminal exon and 46% unknown), involving 10 genes (Table 3).

*CFAP44* (Cilia and Flagella Associated Protein 44) gene shows the higher SI-FC (3.51) with an upregulated SI but, its expression is severely limited to two types of cellular tissues (cilia and flagella); *PLCB2* (1-phosphatidylinositol 4.5-bisphosphate phosphodiesterase β-2) gene had the higher SI-FC (3.23) among downregulated transcripts, but the AS event involved an intron.

*RECK* (Reversion Inducing Cysteine Rich Protein with Kazal Motifs) gene displays a high SI-FC (2.64; *p* = 0.0217), the AS event involves an exon (exon 18) and bibliographic data attest its involvement in extracellular matrix (ECM) homeostasis and inflammation. The splicing event involving exon 18 of *RECK* gene discriminates two different splice variants: the full-length transcript, Long *RECK*, (transcript variant 1; NM_021111) that contains exon 18 and the transcript variant 5, Short *RECK* (NM_001316348) that lacks exon 18.

### 3.2. Clinical Study

Based on RNA-seq results on *RECK* splice variant expression, we choose to evaluate the expression level of Long and Short RECK splice variants in a larger patients’ population. Clinical data of the 128 subjects studied are summarized and reported in Table 4.

Most of the enrolled subjects were male (CTR group 16/24, 66.6%; CAD group 61/72, 84.7%; AMI group 29/32, 90.6%) with age ranged between 33 and 90 years old (CTR median age 67.5 ± 9.3 years old; CAD median age 66.6 ± 9.8; AMI median age 62.2 ± 13). Smoking was an important risk factor in our patients’ group; 17.4% of subjects in CTR group were smokers at the time of the hospitalization compared to 23.9% of patients with stable CAD and to 75% of AMI patients (*p* < 0.0005). The percentage of ex-smokers was higher in CTR and CAD compared to AMI patients (43.5%, 39.4%, and 3.1%, respectively; AMI vs. CTR *p* < 0.0005 and AMI vs. CAD *p* < 0.0005). Five CTR subjects (21.7%) had type 2 diabetes mellitus, whilst twenty-eight CAD patients (39.4%) and nine AMI patients (28.1%). Arterial hypertension was highly represented in all groups, affecting 69.6% of CTR, 77.5% of CAD and 53.1% of AMI patients (AMI vs. CAD *p* < 0.05). Dyslipidemia affected 47.8% of CTR, 88.7% of CAD, and 46.9% of AMI patients (CAD vs. CTR *p* < 0.0005; AMI vs. CAD *p* < 0.0005). We, also, observed that, in both CAD (45.1%) and AMI (46.9%) patients a single vessel disease was more frequent compared to two (29.6% in CAD patients; 28.1% in AMI patients; AMI vs. CTR *p* < 0.005) and three vessels disease (25.4% in CAD patients; 24.2% in AMI patients).

Finally, we observed that the left anterior descending artery (LAD) was the type of vessel more frequently affected (54.9% in CAD and 62.5% in the AMI group) than circumflex artery (CFX) (31% in CAD and 37.6% in the AMI group) and right coronary artery (RCA) (38% in CAD and 5% in the AMI group).

### 3.3. Evaluation of RECK Splice Variants in PBMCs of All Recruited Patients

**Figure 3:**
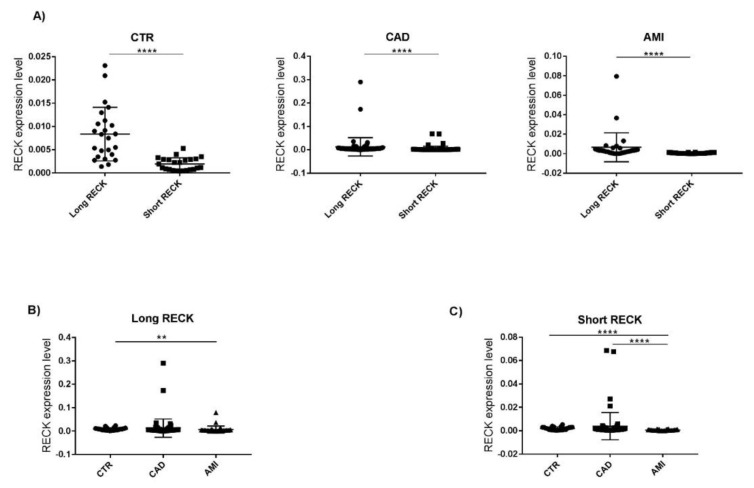
(**A**) Expression level of Long and Short *RECK* splice variants in CTR, CAD, and AMI group. Wilcoxon test, **** *p* < 0.0001. (**B**) Expression level of Long *RECK* in CTR, CAD, and AMI groups. Kruskal–Wallis test, ** *p* < 0.001. (**C**) Expression level of Short *RECK* in CTR, CAD, and AMI groups. Kruskal–Wallis test, **** *p* <0.0001. Expression data are represented as mean ± SD.

The two *RECK* splice variants (Long and Short) have a different expression level in PBMCs of patients. In all three groups the Long splice variant expression level is higher than the Short splice variant (CTR group *p* < 0.0001; CAD group *p* < 0.0001; AMI group *p* < 0.0001) (Figure 3A).

However, Long *RECK* splice variant level is higher in CTR compared to CAD and AMI; interestingly a significant Long *RECK* low expression was observed in AMI vs. CTR (FC = −3.3; *p* < 0.005) (Figure 3B). Analyzing the expression level of the Short splice variant, we observed a significant downregulation in the AMI group both compared both to CTR subjects (FC = −4.5; *p* < 0.0001) and to CAD patients (FC = −4.2; *p* < 0.0001) (Figure 3C).

**Figure 4:**
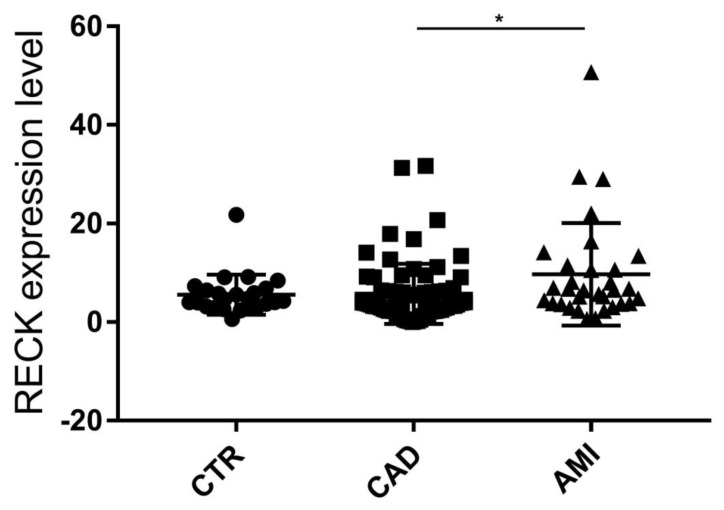
Ratio Long/Short *RECK* splice variants expression level in CTR, CAD, and AMI groups. Kruskal–Wallis test; * *p* < 0.05. Expression data are represented as mean ± SD.

We analyzed the ratio between Long and Short splice variants expression level (Long/Short) in CTR subjects (mean = 5.6 ± 4.1 SD), CAD (mean = 5.7 ± 6.1 SD), and AMI (mean = 9.7 ± 10.4 SD) patients. Noteworthy Long/Short ratio is similar in stable subjects (CTR and CAD). In AMI Long/Short ratio is significantly higher vs. CAD (Figure 4).

### 3.4. ROC Curve

**Figure 5:**
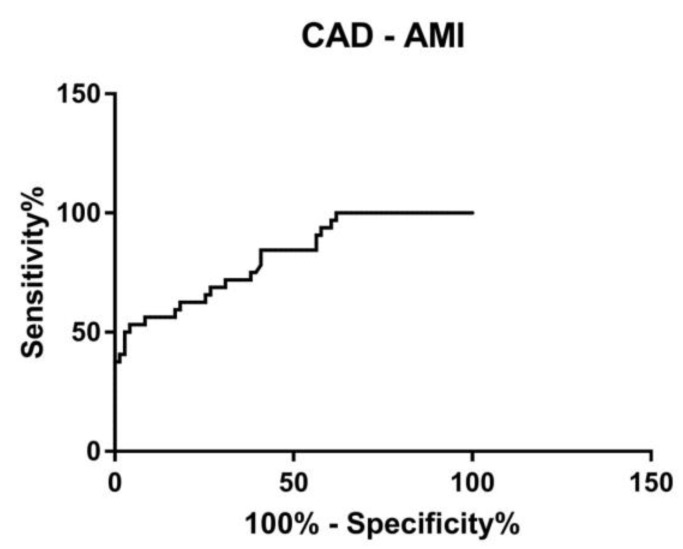
Receiver operator characteristic (ROC) analysis by combining Long and Short *RECK* expression levels. Area under the ROC curve (AUC) = 0.81, 95% confidence interval 0.73 to 0.90, *p* < 0.001.

We conducted a receiver operating characteristic analyses (ROC) to evaluate the potentiality of circulating *RECK* splice variants in identifying patients with stable coronary artery disease (CAD) from those who have an increased risk of developing myocardial event (AMI). We tested a predictive model combining the expression levels of Long and Short splice variant. A combined ROC curve revealed an area under the curve (AUC) of 0.81, with 95% confidence interval 0.73 to 0.90 (*p* < 0.001) between stable CAD and unstable CAD patients (AMI patients) (Figure 5).

### 3.5. Correlation Analysis

**Figure 6:**
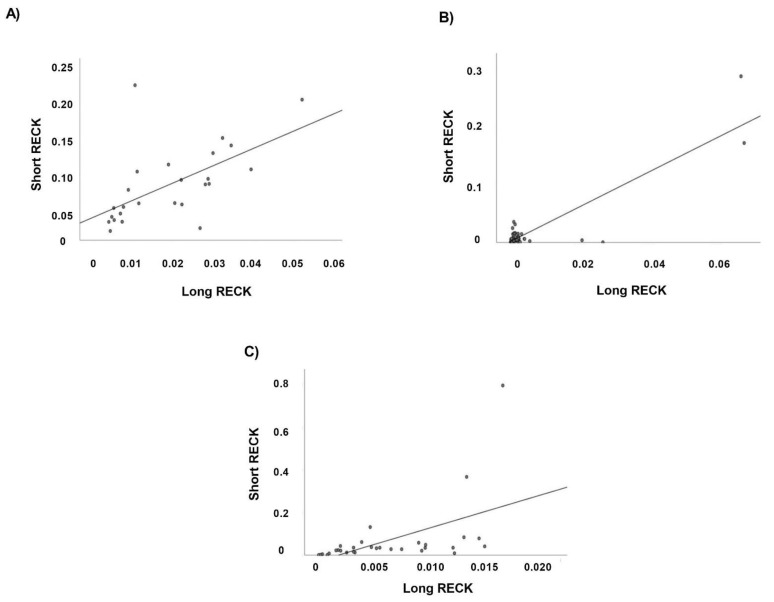
Correlation analysis of Long and Short *RECK* splice variants in all case study. Scatter plots depict the relationship between the two *RECK* splice variants in (**A**) CTR (*R*^2^ = 0.355, *p* < 0.005, Pearson *r* = 0.601); (**B**) CAD (*R*^2^ = 0.793, *p* < 0.0001, Pearson *r* = 0.890); (**C**) AMI (*R*^2^ = 0.279, *p* < 0.005, Pearson *r* = 0.528).

We analyze, by Pearson correlation test, the relationship between the two *RECK* splice variants expression levels in CTR, CAD, and AMI groups. We found a significant positive association between Long and Short *RECK* mRNA in all patients’ groups (Figure 6).

**Figure 7:**
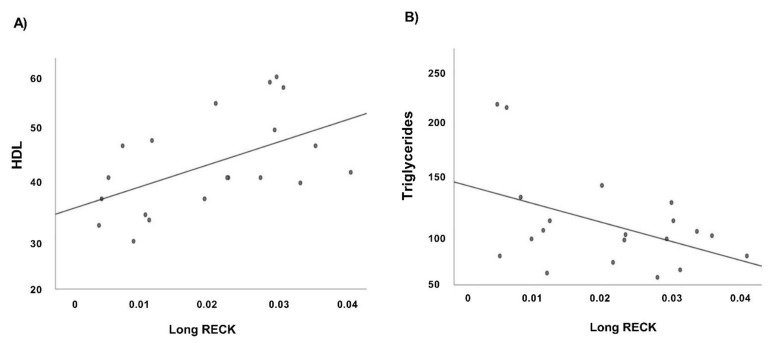
Correlation analysis of Long *RECK* splice variant in CTR group (*n* = 19). Scatter plots depict the relationship among Long *RECK* and (**A**) HDL value (*R*^2^ = 0.297, *p* < 0.05, Pearson *r* = 0.545); (**B**) Triglycerides levels (*R*^2^ = 0.206, *p* < 0.05, Pearson *r* = −0.460).

Moreover, we evaluate the relationship among *RECK* splice variants expression values and the available clinical data of our case study. Regression analyses performed in CTR group show a significant positive correlation between Long *RECK* expression level and HDL values (Figure 7A) and a significant negative correlation with triglycerides (Figure 7B).

**Figure 8:**
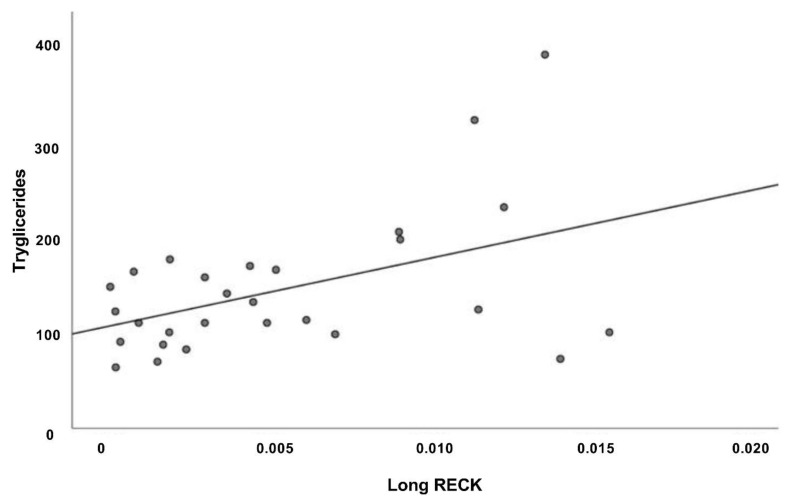
Correlation analysis of Long splice variant in the AMI group (*n* = 28). Scatter plot depicts the relationship among Long *RECK* splice variant and triglycerides levels (*R*^2^ = 0.213, *p* < 0.05, Pearson *r* = 0.461).

The same analysis performed in the AMI group reveal a significant positive correlation between Long splice variant expression level and triglycerides (Figure 8).

No significant correlation between *RECK* splice variants expression level and clinical data was found in CAD patients.

## 4. Discussion

Cardiovascular diseases are a set of heterogeneous diseases of the heart and circulatory system, due to development of atherosclerosis that include CAD and AMI [37,38]. They are the leading cause of premature mortality across the world and in Europe, they cause 49% of mortality [39]. CVDs burden related to modifiable risk factors continues to increase worldwide. High systolic blood pressure, high fasting plasma glucose, high LDL-c levels, tobacco, high BMI, impaired kidney function, impaired diet, low physical activity are the major cardiovascular modifiable risk factors involved in CAD [40]. Moreover, several differences in development and progression of CAD depend on non-modifiable (i.e., age, gender, genetic heritage) risk factors [38,39,41]. Among these, alternative splicing (AS) process represents one important layer of gene expression involved in different complex biological processes [6,42]. Indeed, in the heart, the AS of pre-mRNAs translating sarcomeric proteins, ion channels, and cell signaling proteins is implicated in cardiac development, contractility regulation, and development of different cardiac-related diseases [6]. Moreover, different protein isoforms and splice variants have different biological actions and are involved in various complex processes [43]. This strongly suggests the importance of different protein isoforms. It is fundamental to understand the effects of biological diversity due to the expression of various protein isoforms in our life. Linking the identification of a particular protein isoform to a disease state is one important step that must be followed by the identification of the functional consequences.

This pilot study is based on RNA-seq approach to unravel the AS events in circulating PBMCs of subjects with the absence of atheromatous plaques in coronary arteries (CTR group, *n* = 2) and in patients with stable coronary artery disease (CAD group, *n* = 2) to identify putative genomic biomarkers for risk stratification.

This approach allowed to identify a differential expression pattern and level of two splice variant (Long and Short) of *RECK* gene (Reversion Inducing Cysteine Rich Protein with Kazal Motifs).

*RECK* was first described, in a murine fibroblast cell, as a tumor suppressor acting in the reversion of the malignant phenotype induced by the Ras oncogene [44,45].

The encoded product of *RECK* gene is a 971-residue membrane-anchored glycoprotein of ~125  kDa, which contains an N-terminal signal peptide for secretion, a region spanning five cysteine knots (KNs; KN1-KN5), a region with three repeats like Kazal inhibitors of serine endopeptidases (KLs; KL1-KL3), and a C-terminal segment (CTS; residues A943-N971) [20,46,47,48]. The main function of RECK protein is to regulate cell migration by binding with matrix metalloproteinases so acting as a crucial regulator of extracellular matrix remodeling and signaling pathway [49]. The binding between RECK and MMPs inhibits the conversion of matrix metalloproteinases into their active, matrix-degrading form and alter the degradation of the extracellular matrix (ECM) thus affecting invasive and migratory processes mediated by MMPs [26]. In mice, *Reck* plays an essential role in embryonic and placental vascular remodeling, and these activities have been ascribed to its role as an MMP inhibitor [50]. Mice *Reck*^−/−^ die in utero exhibiting vascular developmental defects and massive hemorrhages. However, in knockout mice for other matrix metalloproteinase inhibitors, these defects were not observed. These results showed an exclusive role of RECK in vascular development and maturation [49]. To date, the exact role of RECK in these processes has not been clarified, but it is hypothesized that RECK plays a key role in the physiology and pathophysiology of angiogenic processes.

RECK attenuates the fibrotic phenotype of cardiac fibroblasts and VSMC [20]. In physiological conditions, RECK is expressed in various organs and cells, but its expression is markedly suppressed in many diseases that promote remodeling. In fact, in these conditions, the upregulation of proteases leads to the release of both membrane-bound and ECM-sequestered growth factors, as well as the breakdown of collagen, and points to adverse cardiovascular remodeling.

Taking these data into consideration, RECK was fundamental in a strategy aimed to inhibit the function of multiple pro-hypertrophic, pro-fibrotic, and proinflammatory mediators to blunt adverse cardiac and vascular remodeling.

*RECK* gene is known to undergo alternative splicing events leading to the formation of five alternative transcripts. Among these alternative transcripts, transcript variant 3 behaves a metastasis-facilitating role, by yet unknown mechanisms; for example, its overexpression promotes adhesion-free growth of glioma cells [25,26]. Moreover, this alternatively spliced *RECK* variant appear to have opposing effects compared to canonical *RECK* (Long *RECK*) and may serve as prognostic markers in glioblastoma patients [25].

Transcript variant 5 (Short *RECK*), analyzed in this study, is highly expressed in proliferating and TGF-β-treated cells [20,21,22]. Short *RECK*, identified by Lee et al. [22] is generated via a combination of alternative splicing and alternative polyadenylation. In fact, Short *RECK* transcript includes a 3′ UTR that is eliminated via splicing from the long *RECK* transcript [22]. At the best of our knowledge, no splicing factor was associated with the events leading to Short *RECK* expression.

Short RECK is involved in cell motility characteristic of cardiovascular processes. Indeed, it has been demonstrated that this transcript variant interacts with the full-length RECK protein (Long *RECK*) to antagonize the inhibition of MMP-9 and favor cellular migration and invasion [21,22]. As reported by Lee et al. [21,22] the interaction between Short RECK and MMP-9 is characteristic and, also, crucial for cell motility; moreover, Trombetta-Lima et al. [25] showed that this important interaction with the MMP-9 is not present for the transcript variant 3, indeed the mRNA expression level of the transcript variant 3 not correlates with MMP-9.

The alteration of MMPs-Long RECK interaction and the consequent release of MMPs, an enhanced expression and/or activation of MMPs, and the alteration of ECM components, are all events associated with the pathogenesis of cardiovascular diseases.

For these reasons, we can hypothesize that the transcript variant 5, that have opposing effects compared to the full-length transcript (Long *RECK*), may have an important role as a biomarker of coronary artery disease.

Our results indicate a differential expression level *RECK* splice variants in all patients’ group analyzed (CTR, CAD, and AMI) (Figure 3).

Patients’ ages were comparable in the three groups analyzed and most of the enrolled subjects were male (CTR group 66.6%; CAD group 84.7%; AMI group 90.6%). However, the prevalence of male gender was significantly higher in CAD and AMI vs. CTR (*p* < 0.05). This data is compatible with the fact that the incidence of CVDs is known to be higher in men than in women of similar age. The different impact of CVDs in men and women probably relates to various factors, including the variable impact of cardiovascular risk factors, different response to therapy, differential gene expression and hormonal factors. Androgens and estrogens influence vascular biology: estrogens have beneficial effects on the cardiovascular system in both men and women, while androgens’ effects depend upon estrogens’ levels in women and upon their aromatization into estrogens in men [51].

Most patients in CAD and AMI groups were smokers or ex-smokers. Cigarette smoking is a major contributor to the risk of AMI and the subsequent morbidity and mortality risks. The prevalence of current smokers was statistically significantly higher in the AMI group than in CTR subjects (*p* < 0.0005) and in CAD patients (*p* < 0.0005). The rate of ex-smokers was statistically significantly lower in AMI patients compared both to CTR subjects (*p* < 0.0005) and to CAD patients (*p* < 0.0005). Current smokers have a higher long-term mortality risk, lower life expectancies and large numbers of life-years lost after AMI [52]. In our case study, dyslipidemia affected 47.8% of CTR subjects, 88.7% of CAD patients and 46.9% of AMI patients, and there was a statistically higher prevalence of dyslipidemia in CAD patients compared to CTR subjects (*p* < 0.0005) and in the comparison AMI vs. CAD patients (*p* < 0.0005). CAD has been directly linked to hypercholesterolemia, especially elevated LDL-c levels. Our results may be explained with the modification of the lipid profile that happens early after an acute coronary syndrome (ACS). Almost all researchers agree that after an AMI a phasic fluctuation of the lipid profile may be observed, with a trend that includes reduced total cholesterol, LDL and HDL and increased triglycerides [53]. These changes generally become manifest within 24–48 h after an acute coronary syndrome, reach a maximum level within approximately 4–7 days and are present for a few months. The mechanisms involved in lipid modification following an ACS are related to the inflammatory acute phase response, the adrenergic-mediated adipocyte lipolysis, the drugs used during hospitalization and the reduction of saturated fat intake during hospitalization [54].

The prevalence of diabetes mellitus was 21.7% in the CTR group, 39.4% in CAD patients and 28.1% in the AMI group and there were no statistically significant differences between the three groups.

Most patients of CTR (69.6%), CAD (77.5%), and AMI (53.1%) groups were affected by arterial hypertension, with a statistically significant higher prevalence of hypertensive subjects in CAD patients than in AMI patients (*p* < 0.05). Arterial hypertension is a major cardiovascular risk factor, with a well-established association with CAD and ACS [55,56].

Most patients of CAD and AMI groups had a one vessel disease (45.1% of CAD and 46.9% of AMI patients), or a two-vessels disease (29.6% of CAD and 28.1% of AMI patients), and the rest of them had a three-vessels disease (25.4% of CAD and 24.2% of AMI patients). In both CAD and AMI patients the most frequent vessel affected was the LAD artery (54.9% and 62.5%, respectively), followed by the CFX artery (31% of CAD and 37.6% of AMI patients) and by the RCA (38% of CAD and 5% of AMI, *p* < 0.0005). The LAD is the most frequently affected vessel in ACS [57,58,59,60].

We observed a significant downregulation of Long *RECK* in AMI patients vs. CTR subjects (Figure 3). This data is in agreement with the functional role of this splice splice variant. Indeed, its lower expression level in AMI patients’ group might indicate a reduction of the interaction with matrix metalloproteinases, in particular MMP9, and consequently changes in ECM composition and promotion of aberrant cellular proliferation. Detailed functional studies on MMP9 will help the understanding of the role of Long/Short *RECK*-MMPs pathway in the progression of atherosclerotic process and contribution to plaque vulnerability.

Moreover, regression analyses indicate a significant positive correlation among Long *RECK* expression level and HDL values in CTR group (Figure 7A). High-density lipoprotein (HDL) cholesterol levels in plasma are inversely associated by the risk of cardiovascular diseases. HDL-c seems to promote cellular cholesterol efflux and to have antioxidant and anti-inflammatory properties. This result may indicate that high Long *RECK* expression level renders patients less prone to develop atherosclerotic plaque and agrees with the published functional data that reveal RECK acting as a MMP inhibitor and also exerting anti-inflammatory effects [61]. Moreover, we observed a significantly negative correlation between Long *RECK* expression level and triglycerides in CTR group (Figure 7B) and a significantly positive correlation in the AMI group (Figure 8).

Elevated triglycerides (TG) levels are considered a risk factor for atherosclerotic cardiovascular disease [62] and elevated fasting and non-fasting TG levels are associated with an increased risk of cardiovascular diseases events in multiple large observational, epidemiological, genetic, and Mendelian randomization studies [63]. Elevated TG levels often accompany elevated LDL-c, low HDL-c levels, diabetes mellitus and metabolic syndrome, so it is difficult to isolate the effect of elevated TG on CVD risk. Current guidelines recommend hypertriglyceridemia management on the basis of fasting triglycerides levels.

The above correlation data support again a protective role of Long *RECK* in atherosclerosis.

When we analyzed the ratio between Long and Short *RECK* expression level, we noticed that it is similar in CTR and CAD groups, i.e., “stable” patients” while it is significantly higher in the comparison AMI vs. CAD. This data suggests that a fine regulation of the opposing Long and Short *RECK* expression might have an active role in the progression of atherosclerosis and in plaque vulnerability.

Due to the differential expression of Long and Short *RECK*, we evaluate their potential to discriminate stable (CAD) from unstable (AMI) patients by a ROC curve analysis. We combined the expression levels of both splice variants and we observed that they allow us to distinguish CAD from AMI patients with an accuracy of 81% (Figure 5). This result supports the hypothesis that a combined evaluation of Long and Short *RECK* expression levels could be a potential good genomic biomarker able to discriminate vulnerable patients (AMI) from non-vulnerable ones (CAD).

## 5. Conclusions

Although this study was carefully prepared, we are aware of some limitations and shortcomings. First, the sample size analyzed in this pilot study is not broad. However, we focused this study only on patients enrolled before the COVID-19 pandemic to eliminate confusing effect due to viral infection. Second, we only examined the expression of the two splice variants of *RECK* gene at the RNA level; consequently, we cannot explicitly conclude that these proteins are differentially expressed in CAD and AMI patients.

Anyway, the different regulation of Long and Short *RECK* splice variants in CTR subjects, CAD and AMI patients indicate that their expression level might be a good genomic biomarker of clinical outcome in coronary artery disease patients. Finally, the low expression of Long *RECK* in AMI and the dysregulation of Long/Short ratio in the same patients suggest a protective role of the canonical splice variant, thus supporting previous data that pointed out how a sustained *RECK* expression might have therapeutic potential in inhibiting adverse cardiac and vascular remodeling.

## Figures and Tables

**Table 1:** Real-Time PCR primer sequences and amplification settings.

Genes	Accession Number	Sequence (5′-3′)		Annealing Temperature (°C)	Size (bp)
*RECK* (Short splice variant)	NM_001316348	Fw	GAACAGACTCTTCTCCTGGT	56	208
		Rev	AGATATCAGGCTCTCTTCTCA		
*RECK* (Long splice variant)	NM_021111	Fw	CCAGCCCTTTTGCAGAGCA	60	221
		Rev	AAGCACCCGGTGGGATGAT		
*GAPDH*	NM_002046	Fw	AAGGTCGGAGTCAACGGATTT	59	100
		Rev	TGAAGGGGTCATTGATGGCA		

*RECK* (Reversion Inducing Cysteine Rich Protein with Kazal Motifs), *GAPDH* (Glyceraldehyde 3-phosphate dehydrogenase). Fw (Forward primer), Rev (Reverse primer).

**Table 2:** Number of AS regulated events.

Alternative Event Type	N. of AS Regulated Events
Alternative First Exon	14
Alternative Terminal Exon	10
Exon Cassette	14
Alternative Acceptor Splice Site	5
Alternative Donor Splice Site	6
Intron Retention	6
unknown	58

**Table 3:** 10 differentially regulated AS events in CAD PBMCs involving different 10 genes.

Gene Symbol	Accession Number	Alternative Event Type	Involved Exon	Regulation Splicing Index	Splicing-Index Fold-Change	Splicing-Index *p*-Value	Event Coordinates (hg19)
*CFAP44*	NM_001164496	unknown	e25	up	3.51	4.87 × 10^−2^	chr3:113052252-113052429
*PLCB2*	NM_004573	unknown	i31	down	3.23	9.14 × 10^−4^	chr15:40581119-40581471
*RECK*	NM_021111	unknown	e18	down	2.64	2.17 × 10^−2^	chr9:36118754-36118964
*VTI1A*	NM_145206	unknown	i1	up	2.63	6.80 × 10^−3^	chr10:114208248-114208639
*CD58*	NM_001779	Alternative Terminal Exon	ae5	down	2.45	3.91 × 10^−2^	chr1:117061321-117061851
*MIR4469 // RNF170*	NM_030954	Alternative Terminal Exon	ae6	down	2.29	2.93 × 10^−2^	chr8:42716503-42716886
*PTER*	NM_001001484	Exon Cassette	e2	down	2.21	2.41 × 10^−2^	chr10:16479356-16479489
*GPATCH2L*	NM_017926	Alternative Terminal Exon	ae9	down	2.19	4.61 × 10^−2^	chr14:76662316-76662739
*CLEC12A*	NM_138337	unknown	i1	down	2.13	1.23 × 10^−2^	chr12:10124287-10131564
*BLNK*	NM_013314	Exon Cassette	e16	down	2.13	3.31 × 10^−2^	chr10:97956663-97956735

CFAP44 (Cilia and Flagella Associated Protein 44); PLCB2 (1-phosphatidylinositol 4.5-bisphosphate phosphodiesterase β-2); RECK (Reversion Inducing Cysteine Rich Protein with Kazal Motifs); VTI1A (vesicle transport through interaction with t-SNAREs 1A); RNF170 (ring finger protein 170); PTER (Phosphotriesterase-Related); GPATCH2L (G-patch domain containing 2 like); CLEC12A (C-type lectin domain family 12 member A); BLNK (B Cell Linker).

**Table 4:** Clinical features of CTR, CAD, and AMI patients studied.

	CTR Subjects	CAD Patients	AMI Patients	*p*-Value
Age (years)	67.5 ± 9.3	66.6 ± 9.8	62.2 ± 13	n.s.
Gender				
Male (%)	66.6	84.7	90.6	* *p* < 0.05
				^Ɨ^ *p* < 0.05
Hypertension (%)	69.6	77.5	53.1	^ǂ^ *p* < 0.05
Diabetes (%)	21.7	39.4	28.1	n.s.
Dyslipidemia (%)	47.8	88.7	46.9	* *p* < 0.0005
				^ǂ^ *p* < 0.0005
Smoking history				
Present (%)	17.4	23.9	75	^Ɨ^ *p* < 0.0005
				^ǂ^ *p* < 0.0005
Past (%)	43.5	39.4	3.1	^Ɨ^ *p* < 0.0005
				^ǂ^ *p* < 0.0005
Number of affected vessels				
1 vessel disease (%)	0	45.1	46.9	* *p* < 0.0005
				^Ɨ^ *p* < 0.0005
2 vessel disease (%)	0	29.6	28.1	* *p* < 0.005
				^Ɨ^ *p* < 0.005
3 vessel disease (%)	0	25.4	24.2	* *p* < 0.005
				^Ɨ^ *p* < 0.005
Type of affected vessel				
LAD (%)	0	54.9	62.5	* *p* < 0.0005
				^Ɨ^ *p* < 0.0005
CFX (%)	0	31	37.6	* *p* < 0.0005
				^Ɨ^ *p* < 0.0005
RCA (%)	0	38	5	* *p* < 0.0005
				^ǂ^ *p* < 0.0005

Continuous data are expressed as mean ± SD; categorical data are expressed as percentage. LAD. Left descending artery; CFX. Circumflex coronary artery; RCA. Right coronary artery; 0 = absence. Student’s *t*-test was used to assess significance. * CAD vs. CTR group; ^Ɨ^ AMI vs. CTR group; ^ǂ^ AMI vs. CAD group. n.s. = not significant

## Data Availability

All relevant data are within the manuscript and in the Supporting Information file (GSE173983) publicly available on https://www.ncbi.nlm.nih.gov/geo/query/acc.cgi?acc=GSE173983 accessed on 14 June 2021).

## Supplementary Materials

The following are available online at https://www.mdpi.com/article/10.3390/genes12060939/s1, Figure S1: The human RECK gene spans 87 kb on chromosome region 9p13. (A) Genomic structure of Long *RECK* splice variant. This transcript is 4412 bp long and is constituted by 21 exons. (B) Genomic structure of Short *RECK* splice variant. This transcript is 1833 bp long and is constituted by 9 exons. Grey boxes indicate exons. Table S1: Clinical features of CTR and CAD patients (*n* = 2) selected for sequencing study.
