# Supplementary material for: Two RECK Splice Variants (Long and Short) Are Differentially Expressed in Patients with Stable and Unstable Coronary Artery Disease: A Pilot Study
Source: Genes (Basel). 2021 Jun 19;12(6):939. doi: 10.3390/genes12060939 (PMC8234100; doi:10.3390/genes12060939)
Supplement: Supplementary file 1 [file genes-12-00939-s001.zip › genes-1245661-supplementary.pdf]

SUPPLEMENTARY TABLES AND FIGURES

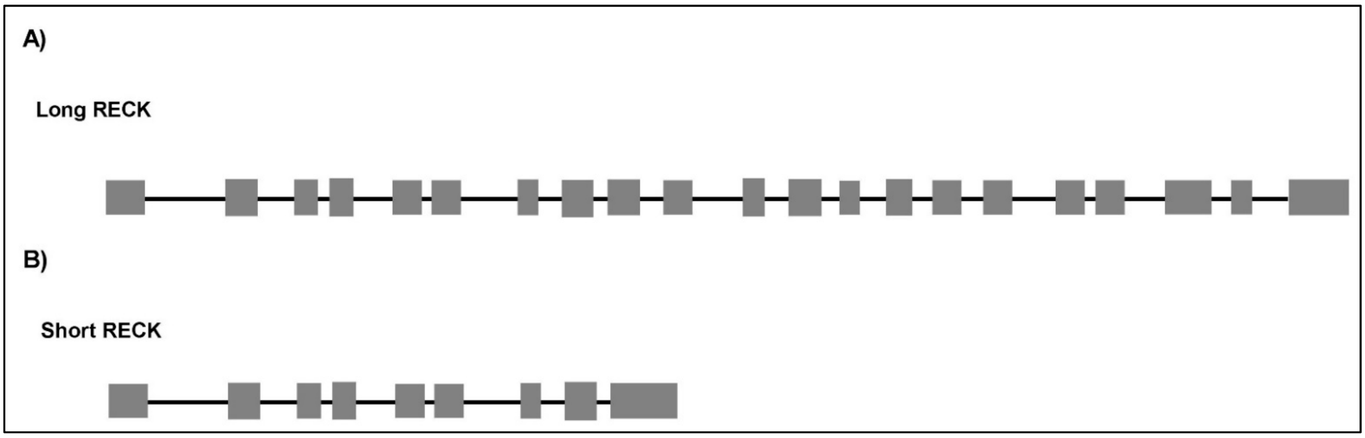

Supplementary Figure S1. The human *RECK* gene spans 87 kb on chromosome region 9p13. (A) Genomic structure of Long *RECK* splice variant. This transcript is 4412 bp long and is constituted by 21 exons. (B) Genomic structure of Short *RECK* splice variant. This transcript is 1833 bp long and is constituted by 9 exons. Grey boxes indicate exons.

Supplementary Table S1. Clinical features of CTR and CAD patients (n=2) selected for sequencing study.

	CTR subjects	CAD patients	*p*-value
Age (years)	70.5 ± 11.1	71.5 ± 4.9	n.s.
Gender			
Male (%)	100	100	n.s.
Hypertension (%)	100	100	n.s.
Diabetes (%)	-	50	n.s.
Dyslipidemia (%)	100	100	n.s.
Smoking history			
Present (%)	-	-	n.s.
Past (%)	-	100	$p < 0.05$
Number of affected vessels			
1 vessel disease (%)	-	50	n.s.
2 vessel disease (%)	-	-	n.s.
3 vessel disease (%)	-	50	n.s.
Type of affected vessel			
LAD (%)	-	50	n.s.
CFX (%)	-	100	$p < 0.05$
RCA (%)	-	50	n.s.

Continuous data are expressed as mean ± SD; categorical data are expressed as percentage. LAD. left descending artery; CFX. circumflex coronary artery; RCA. right coronary artery; - = absence. Student *t*-test was used to assess significance.
